# Albumin-Based Biomarkers and Adverse Outcomes in Coronary Artery Disease: A Systematic Review and Meta-Analysis

**DOI:** 10.3390/jcm15176868

**Published:** 2026-09-04

**Authors:** Yanwu Yang, Tianyi Qu, Yan Zhang, Zhi Wan, Meiling Ge

**Affiliations:** 1 The Emergency Department, West China Hospital, Sichuan University, Chengdu 610041, China; yywatc@wchscu.cn; 2 Laboratory of Cardiac Structure and Function, Institute of Cardiovascular Diseases, West China Hospital, Sichuan University, Chengdu 610041, China; tianyiqu@wchscu.cn; 3 Center of Gerontology and Geriatrics and National Clinical Research Center for Geriatrics, West China Hospital, Sichuan University, Chengdu 610041, China; yanzhang@wchscu.cn

**Keywords:** albumin-based biomarkers, coronary artery disease (CAD), C-reactive protein-to-albumin ratio (CAR), neutrophil percentage-to-albumin ratio (NPAR), lactate-to-albumin ratio (LAR), C-reactive protein–albumin–lymphocyte (CALLY) index

## Abstract

**Background**: Albumin-based biomarkers have emerged as practical tools for prognostic assessment in coronary artery disease (CAD), but their comparative prognostic patterns and potential clinical utility have not been systematically evaluated. **Methods**: We performed a systematic review and meta-analysis of albumin-based biomarkers, including the C-reactive protein-to-albumin ratio (CAR/hsCAR), neutrophil percentage-to-albumin ratio (NPAR), lactate-to-albumin ratio (LAR), and C-reactive protein–albumin–lymphocyte (CALLY) index, in patients with CAD. Pooled associations with mortality and MACE were assessed, together with discriminative performance and clinical utility. **Results**: Thirty-four studies involving 47,763 patients were included. Higher CALLY values were associated with lower all-cause mortality (HR 0.46, 95% CI 0.24–0.87) and a directionally protective but non-significant association with MACE (HR 0.44, 95% CI 0.18–1.08). Elevated CAR/hsCAR was associated with increased mortality (OR 1.61, 95% CI 1.09–2.38) and MACE (HR 1.37, 95% CI 1.21–1.56) in acute coronary syndrome (ACS)-related cohorts. NPAR showed the most consistent association with mortality (HR 1.88, 95% CI 1.71–2.08; I^2^ = 4.8%), whereas its association with MACE was less stable (HR 1.86, 95% CI 0.56–6.13). LAR showed the largest pooled effect estimate for mortality (HR 2.50, 95% CI 1.78–3.51). In discriminative analyses, CAR showed the most balanced performance for long-term MACE, NPAR showed the strongest rule-in profile for long-term MACE, and CAR and CALLY showed the best discrimination for mortality. **Conclusions**: Albumin-based biomarkers are linked to adverse CAD outcomes, especially in ACS, with distinct prognostic patterns by biomarker and endpoint. They provide complementary risk stratification information, and further prospective studies are needed to confirm their incremental clinical utility.

## 1. Introduction

Coronary artery disease (CAD) remains a leading cause of morbidity and mortality worldwide, with acute coronary syndrome (ACS) representing its most unstable clinical presentation [1]. Despite major advances in revascularization, antithrombotic therapy, and secondary prevention, patients with ACS continue to face substantial long-term risks of recurrent myocardial infarction, stroke, heart failure, and cardiovascular death, with considerable heterogeneity in prognosis [2]. This persistent residual risk highlights the need for simple, inexpensive, and readily available tools to improve early prognostic stratification in routine clinical practice, particularly in ACS-related settings.

Serum albumin has attracted increasing interest in cardiovascular risk assessment. Beyond its conventional role as a marker of nutritional and physiological reserve, albumin is a multifunctional protein with antioxidant, anti-inflammatory, endothelial-protective, and anticoagulant properties. During acute or chronic systemic inflammation, inflammatory activation may reduce albumin synthesis and increase vascular permeability, while oxidative stress can impair the antioxidant functions of circulating albumin. Accordingly, hypoalbuminemia may reflect not only impaired nutritional reserve, but also a broader state of inflammatory activation, oxidative imbalance, organ dysfunction, and reduced physiological resilience [3,4]. These abnormalities frequently coexist with endothelial dysfunction and may characterize patients with a greater burden of atherosclerotic and systemic disease, thereby providing a biological basis for the association between low albumin concentrations and adverse coronary outcomes [5].

To better capture these interrelated pathophysiological dimensions, a growing number of albumin-based biomarkers have been proposed. These indices combine albumin with inflammatory, immune, or metabolic variables, including the C-reactive protein-to-albumin ratio (CAR), high-sensitivity CAR (hsCAR), neutrophil percentage-to-albumin ratio (NPAR), lactate-to-albumin ratio (LAR), and the C-reactive protein–albumin–lymphocyte (CALLY) index [6,7,8,9]. Although these biomarkers share albumin as a common component, the accompanying variables reflect biologically distinct processes, including systemic inflammation, innate or adaptive immune activation, and metabolic or hemodynamic stress. Their prognostic relevance may therefore arise from their ability to integrate overlapping but complementary dimensions of cardiovascular vulnerability rather than reflecting nutritional status alone [10].

The literature on these biomarkers has expanded rapidly, but several gaps remain. Most studies have assessed these indices individually, and their prognostic patterns have not been examined within a unified framework. In addition, the available evidence is heterogeneous with respect to study population, biomarker definition, follow-up duration, and endpoint ascertainment. Data on discriminative performance and potential clinical utility are also less well synthesized than association data. At the same time, an exhaustive review of all albumin-related indices is neither practical nor equally informative, because some biomarkers have been rarely studied, whereas others, such as the fibrinogen-to-albumin ratio, have already been reviewed in related coronary settings [11].

We therefore conducted a systematic review and meta-analysis of albumin-based biomarkers, including CAR/hsCAR, NPAR, LAR, and CALLY, to evaluate their associations with adverse outcomes in CAD populations, predominantly in ACS-related settings. We also compared their discriminative performance and explored their potential clinical utility for predicting mortality and major adverse cardiovascular events.

## 2. Methods

### 2.1. Protocol and Literature Search

This systematic review and meta-analysis was conducted in accordance with the PRISMA 2020 guidelines [12,13] (checklist provided in Supplementary Table S1).

A comprehensive literature search was performed independently by two reviewers in PubMed, Embase, Web of Science, and the Cochrane Central Register of Controlled Trials from database inception to March 2026. The search strategy combined subject headings and free-text terms related to coronary artery disease, albumin-based biomarkers, and prognosis. The biomarkers of interest included the CAR/hsCAR, NPAR, LAR, and CALLY index. Outcome-related terms included all-cause mortality, major adverse cardiovascular events, and prognosis (detailed syntax in Supplementary Table S2). In addition, the reference lists of relevant reviews and all included studies were screened manually to identify any potentially eligible articles. No language restriction was applied during the initial search. The present review focused on CAR/hsCAR, NPAR, LAR, and CALLY because these albumin-based indices had an established and sufficiently developed prognostic literature in CAD or ACS populations to support quantitative synthesis and represented complementary inflammatory, immune, nutritional, and metabolic or hemodynamic domains. Albumin-related indices supported by only limited coronary evidence, as well as indices that had already been systematically synthesized in closely related coronary settings, were not prioritized in the present review.

### 2.2. Eligibility Criteria

Studies were considered eligible if they met the following criteria: (1) enrolled adult patients with CAD, including acute coronary syndrome or other clearly defined coronary syndromes; (2) evaluated at least one albumin-based biomarker, including CAR/hsCAR, NPAR, LAR, or CALLY; (3) reported the association between these biomarkers and clinical outcomes; (4) provided effect estimates with corresponding 95% confidence intervals, or sufficient data for their calculation; and (5) used an observational design, including cohort or case–control studies. Studies were excluded if they were reviews, editorials, letters, conference abstracts, case reports, or experimental studies; if they focused on non-coronary cardiovascular conditions without separately extractable coronary data; or if the relevant outcomes could not be obtained from the original report.

### 2.3. Data Extraction and Quality Assessment

After removal of duplicates, titles and abstracts were screened independently by two reviewers, followed by full-text assessment of potentially eligible studies. Disagreements were resolved through discussion, and when necessary, by consultation with a third reviewer. Data were extracted independently using a standardized form. The following information was collected from each study: first author, year of publication, country, study design, sample size, population characteristics, clinical setting, follow-up duration, type of albumin-based biomarker, cut-off value or exposure model, outcome definitions, adjustment variables, and effect estimates with 95% CIs. When multiple models were reported, the estimate derived from the most fully adjusted model was extracted for the primary analysis.

The methodological quality of the included cohort studies was evaluated using the Newcastle–Ottawa Scale (NOS). Studies scoring ≥ 7 stars out of a maximum of 9 were graded as high quality. Any discrepancies arising during the literature selection, data extraction, or quality appraisal phases were resolved through consensus or arbitration by a third senior investigator.

### 2.4. Statistical Analysis

Pooled analyses were performed separately for each biomarker–outcome pair, and studies were combined only when they evaluated the same biomarker and clinically comparable outcomes. Mortality outcomes and major adverse cardiovascular event outcomes were analyzed separately. Because the included studies used different cutoff values and exposure-grouping strategies, study-specific definitions were retained rather than converted to a common threshold; the pooled estimates therefore represent relative prognostic associations across the reported exposure contrasts rather than effects at a universal clinical cutoff. Differences in study population and follow-up duration were explored using subgroup or restricted analyses where sufficient data were available. Given the anticipated variability in clinical setting, follow-up, biomarker definition, and outcome ascertainment, random-effects models were primarily used to incorporate residual between-study heterogeneity. Between-study heterogeneity was assessed using Cochran’s Q test and the I^2^ statistic. Potential sources of heterogeneity were explored using subgroup analyses according to population type, biomarker subtype, and follow-up duration when sufficient data were available. Leave-one-out sensitivity analyses were performed for pooled outcomes including at least four studies to assess the influence of individual studies on the summary estimates. Publication bias was planned to be assessed using funnel plots and Egger’s regression test when at least 10 studies were available for a pooled outcome; however, none of the analyses met this threshold.

Subgroup analyses were conducted according to population type, biomarker subtype, and follow-up duration where data permitted. Leave-one-out sensitivity analyses were performed for pooled outcomes including at least four studies. Publication bias was planned to be assessed by funnel plots and Egger’s regression test when at least 10 studies were available; however, no outcome met this threshold. For biomarkers with sufficient data, discriminative performance was evaluated using bivariate hierarchical models to estimate pooled sensitivity, specificity, and SROC curves, with the area under the curve (AUC) summarizing overall discrimination. Clinical utility was explored using Fagan’s nomograms. All analyses were performed using R software, (version 4.4.2; R Foundation for Statistical Computing, Vienna, Austria) mainly with the meta and mada packages. A two-sided *p*-value < 0.05 was considered statistically significant.

## 3. Results

### 3.1. Study Characteristics

**Figure 1:**
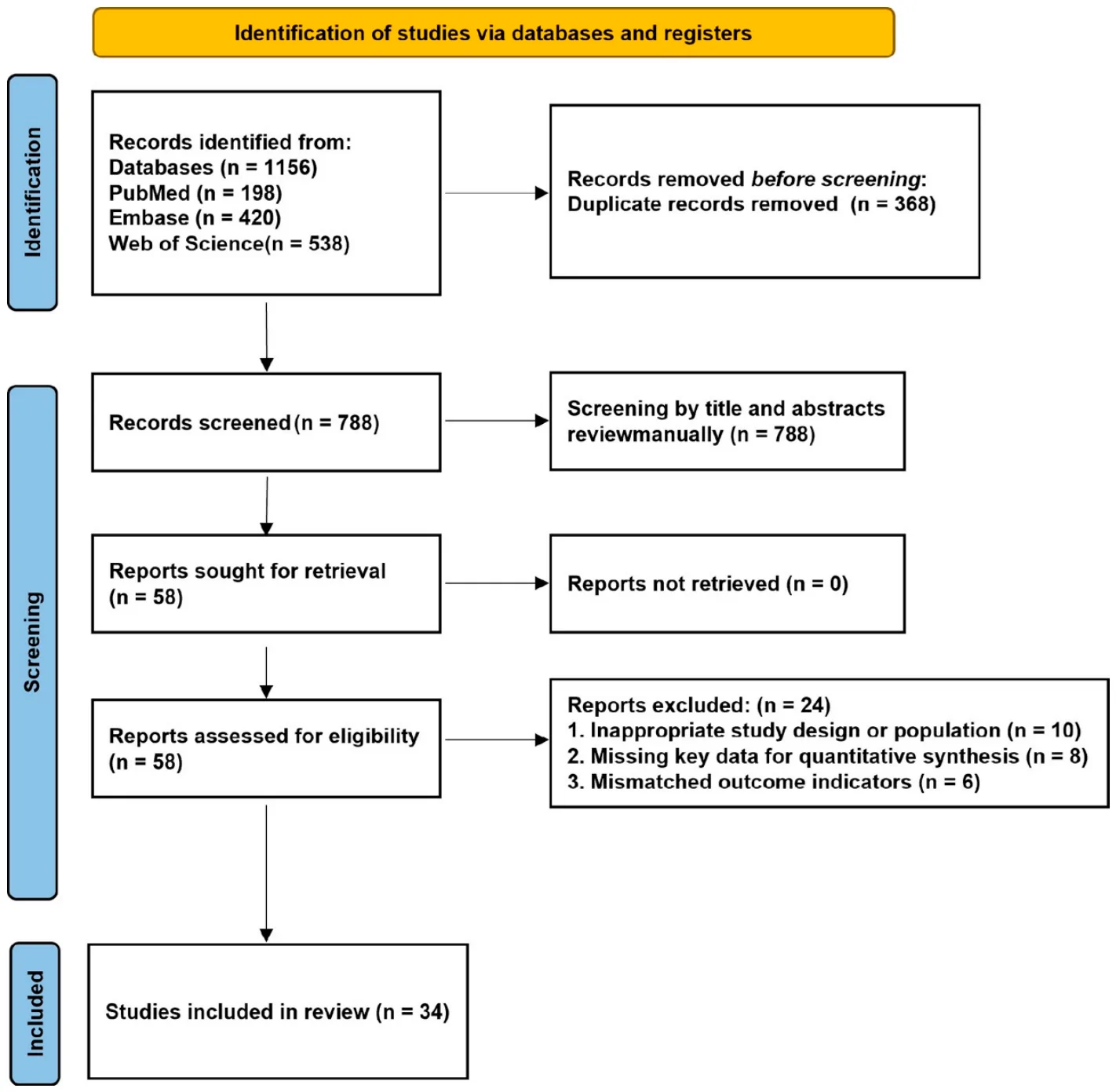
PRISMA flow diagram of study selection.

The initial systematic literature search yielded a total of 1156 records across the searched databases. After the removal of 368 duplicate records and screening of titles and abstracts, 58 articles were retrieved for full-text evaluation. Ultimately, 34 independent observational studies met the predefined inclusion criteria and were synthesized in this meta-analysis [14,15,16,17,18,19,20,21,22,23,24,25,26,27,28,29,30,31,32,33,34,35,36,37,38,39,40,41,42,43,44,45,46,47]. The detailed step-by-step screening process is illustrated in the PRISMA flow diagram (Figure 1).

A total of 34 studies published between 2019 and 2026 were included, comprising 47,763 patients. Most studies were conducted in China, with the remainder from Turkey, and all but one used a retrospective design. Sample sizes ranged from 116 to 4313 patients (Table 1). Overall, the included populations were within the CAD spectrum and were predominantly ACS-related, including STEMI, NSTEMI, AMI, and mixed ACS cohorts. Several studies specifically enrolled patients undergoing PCI, whereas a smaller number included broader CAD or CHD populations. Across studies, the mean age was generally between the mid-50s and early 70s, and most cohorts were predominantly male. The biomarkers evaluated were CAR/hsCAR (*n* = 15), NPAR (*n* = 8), LAR (*n* = 6), and CALLY (*n* = 7), with CAR/hsCAR being the most frequently studied. Biomarker definitions varied across studies: some used prespecified cutoffs, whereas others analyzed biomarkers as dichotomized variables, tertiles, or quartiles. Follow-up ranged from in-hospital assessment to 122 months. The main outcomes were all-cause mortality, cardiovascular mortality, major adverse cardiovascular events (MACE), and major adverse cardiac and cerebrovascular events (MACCE), although several studies also reported time-specific mortality endpoints such as 28-day, 30-day, or 1-year mortality. Methodological quality was generally high. Based on the Newcastle Ottawa Scale, all included cohorts scored between 6 and 9 (Supplementary Table S3). Minor score reductions were mainly related to limited multivariable adjustment or short follow-up in studies restricted to in-hospital outcomes. No study was classified as high risk of bias.

### 3.2. Prognostic Impact of Albumin-Based Biomarkers

**Figure 2:**
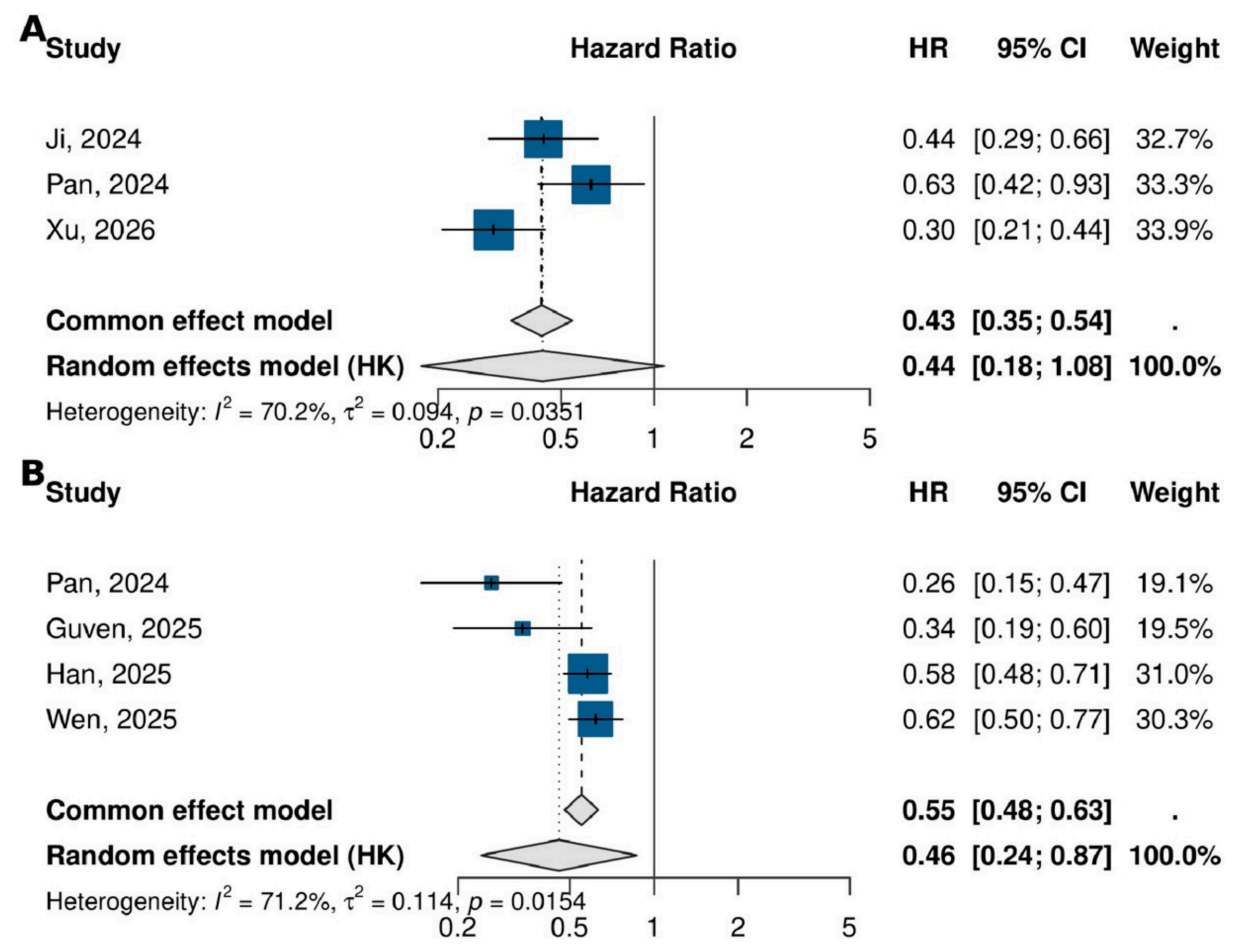
Forest plots of the association between the C-reactive protein–albumin–lymphocyte (CALLY) index and adverse outcomes. (**A**) Association between the CALLY index and major adverse cardiovascular events (MACE) [27,28,33]. (**B**) Association between the CALLY index and all-cause mortality [28,30,31,32].

Higher CALLY was associated with lower risks of both MACE and all-cause mortality. For MACE, pooled analysis of three studies involving 4658 patients showed a protective but non-significant association (HR: 0.44, 95% CI: 0.18–1.08; I^2^ = 70.2%). For all-cause mortality, the association was statistically significant across four studies including 10,800 patients (HR: 0.46, 95% CI: 0.24–0.87; I^2^ = 71.2%) (Figure 2).

**Figure 3:**
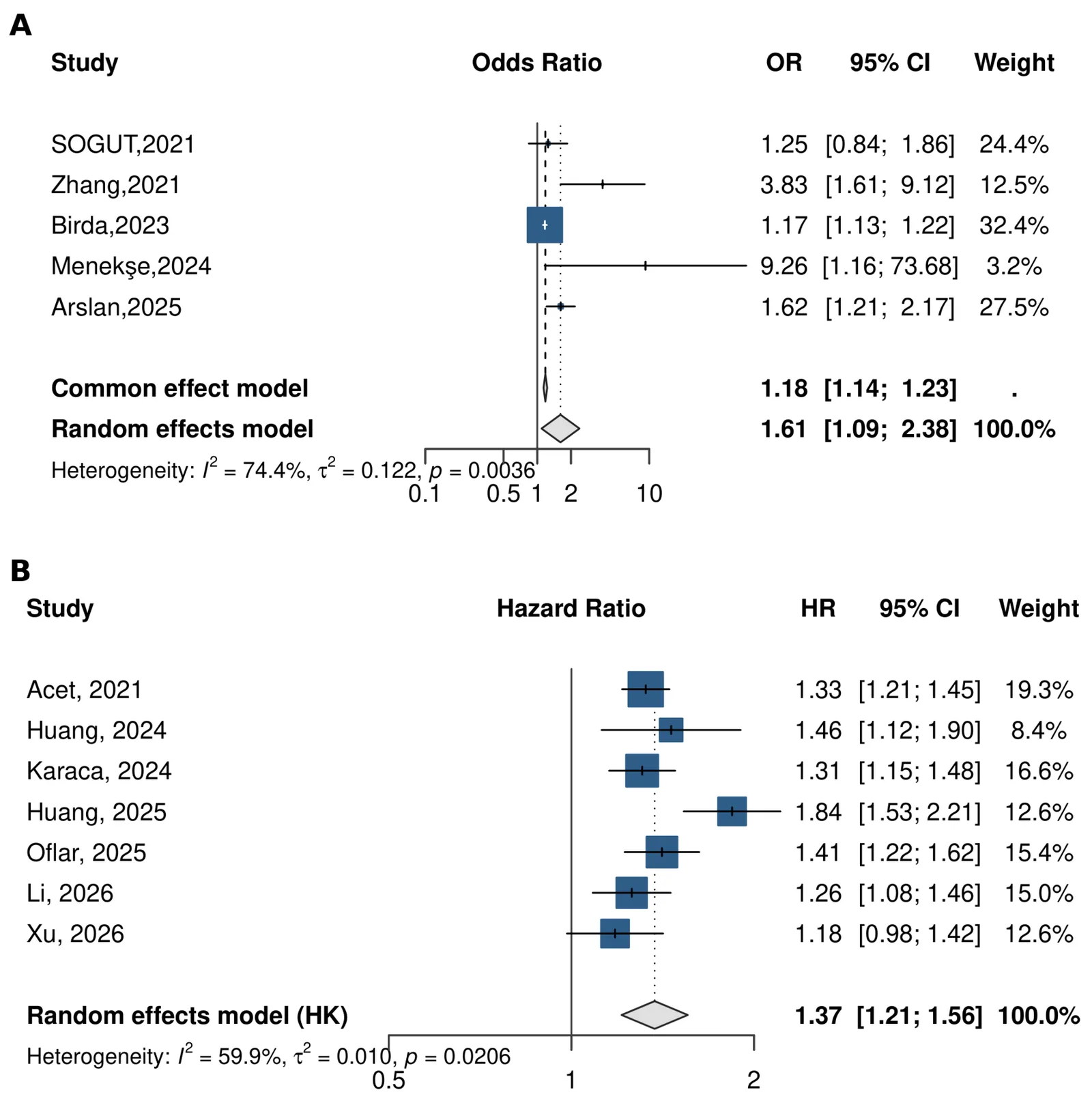
Forest plots of the association between the C-reactive protein-to-albumin ratio/high-sensitivity C-reactive protein-to-albumin ratio (CAR/hsCAR) and adverse outcomes. (**A**) Association between CAR/hsCAR and mortality, expressed as pooled odds ratios (ORs) [17,18,19,22,23]. (**B**) Association between CAR/hsCAR and major adverse cardiovascular events (MACE), expressed as pooled hazard ratios (HRs) [16,21,24,25,26,33,37].

In contrast, higher CAR/hsCAR was consistently associated with adverse outcomes in patients with ACS (Figure 3). Across five studies including 4522 patients, elevated CAR/hsCAR was associated with increased mortality risk (OR: 1.61, 95% CI: 1.09–2.38; I^2^ = 74.4%). Similarly, pooled analysis of seven studies involving 5194 patients showed a significant association with MACE (HR: 1.37, 95% CI: 1.21–1.56; I^2^ = 59.9%). In subgroup analyses for MACE, the pooled HR was 1.32 (95% CI: 1.21–1.44; I^2^ = 0.0%) in studies using conventional CAR and 1.50 (95% CI: 0.91–2.45; I^2^ = 80.0%) in those using hsCAR, suggesting greater estimate stability for conventional CAR, although the subgroup difference was not significant (Figure S1).

**Figure 4:**
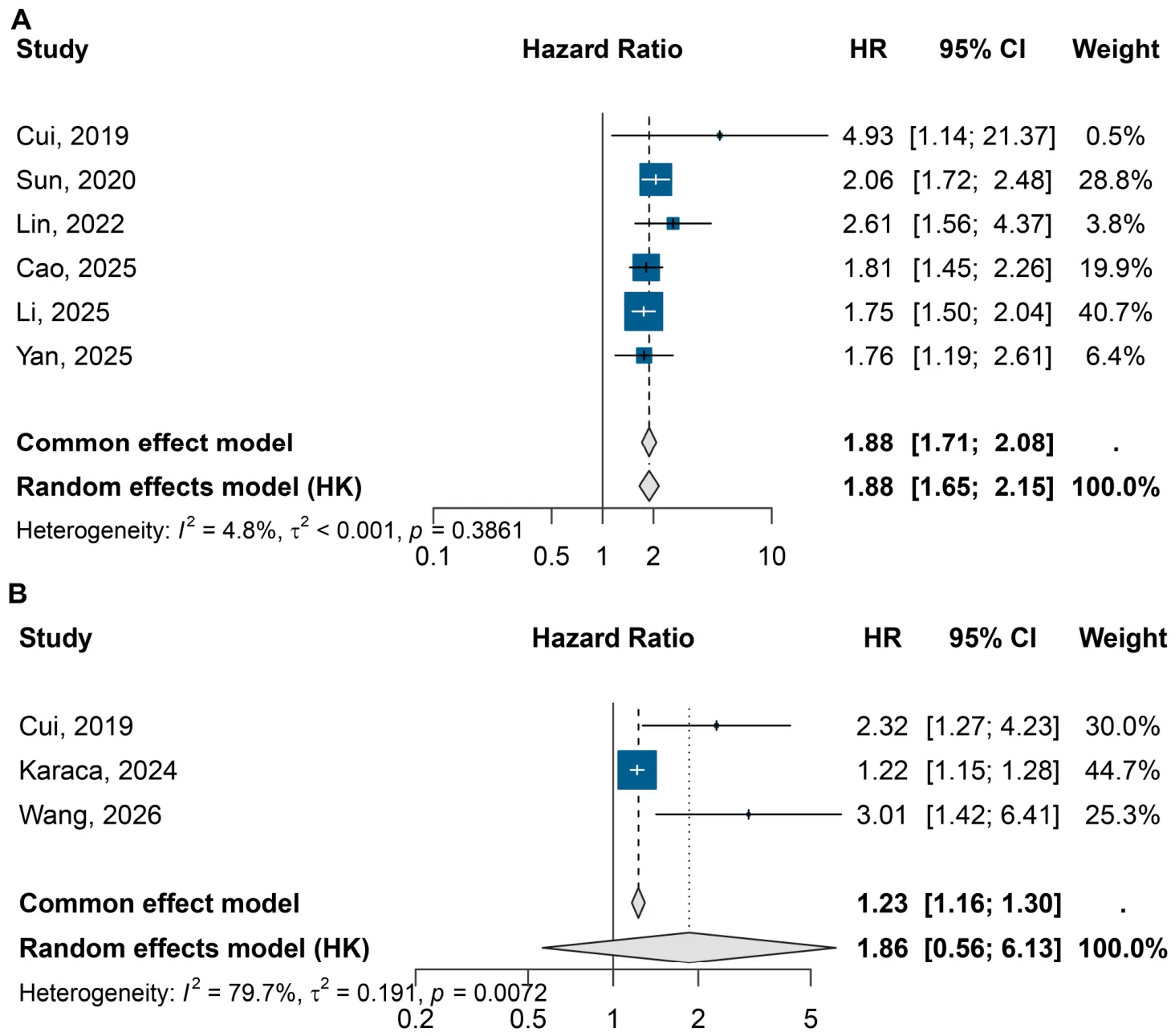
Forest plots of the association between the neutrophil percentage-to-albumin ratio (NPAR) and adverse outcomes. (**A**) Association between NPAR and mortality [34,35,36,38,39,40]. (**B**) Association between NPAR and major adverse cardiovascular events (MACE) [34,37,41].

NPAR showed the most consistent association with mortality (Figure 4). In the overall pooled analysis of six studies including 10,785 patients, elevated NPAR was associated with increased mortality risk (HR: 1.88, 95% CI: 1.71–2.08; I^2^ = 4.8%). This association remained stable in both long-term mortality (HR: 1.88, 95% CI: 1.70–2.07; I^2^ = 0.0%) and short-term mortality (HR: 1.97, 95% CI: 1.63–2.39; I^2^ = 0.0%) (Figure S2). Additional subgroup analysis by population type showed generally consistent associations across both ACS-related and broader CAD cohorts (Figure S3). In ACS-related cohorts, the common-effect estimate was 1.95 (95% CI: 1.59–2.39; I^2^ = 37.5%), whereas in broader CAD cohorts the pooled HR was 1.86 (95% CI: 1.66–2.09; I^2^ = 0.0%), with no significant subgroup difference. For MACE, however, the pooled estimate from three studies involving 3021 patients was not statistically significant and showed substantial heterogeneity (HR: 1.86, 95% CI: 0.56–6.13; I^2^ = 79.7%).

**Figure 5:**
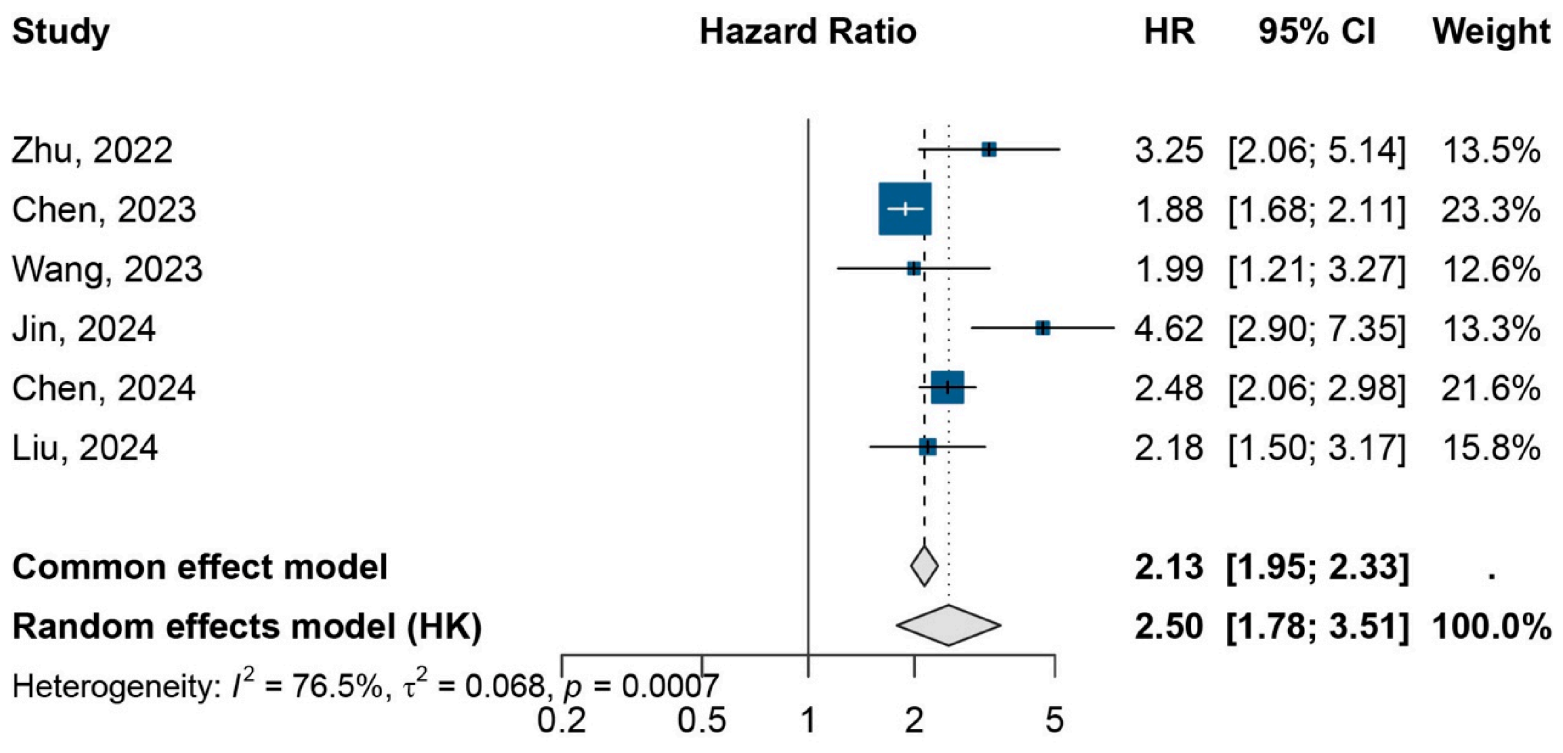
Forest plot of the association between the lactate-to-albumin ratio (LAR) and mortality [42,43,44,45,46,47].

Higher LAR was also associated with increased mortality risk. Across six studies including 10,310 patients, the pooled HR was 2.50 (95% CI: 1.78–3.51; I^2^ = 76.5%), representing the largest mortality effect estimate among the included biomarkers (Figure 5). To improve population homogeneity, an additional analysis restricted to ACS-related cohorts was performed, and the association remained significant (HR: 2.59, 95% CI: 1.66–4.04; I^2^ = 81.8%) (Figure S4).

**Figure 6:**
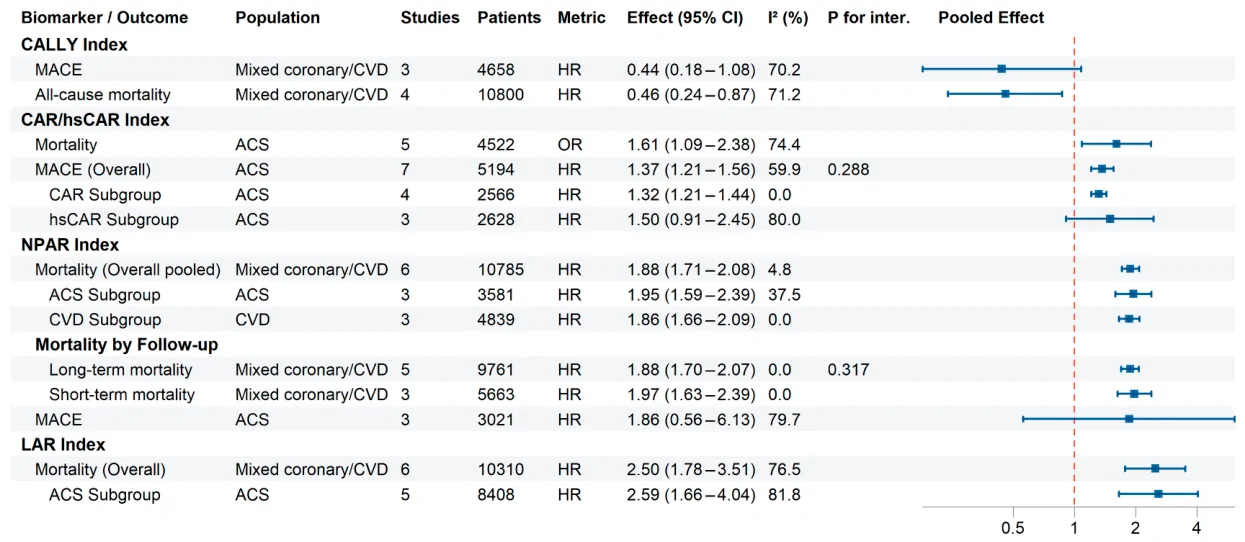
Summary of the pooled prognostic associations of selected albumin-based composite biomarkers with adverse outcomes. Abbreviations: CAR, C-reactive protein-to-albumin ratio; hsCAR, high-sensitivity C-reactive protein-to-albumin ratio; NPAR, neutrophil percentage-to-albumin ratio; LAR, lactate-to-albumin ratio; CALLY, C-reactive protein–albumin–lymphocyte index; MACE, major adverse cardiovascular events. Points and horizontal lines represent pooled estimates and 95% CIs, respectively; the dashed vertical line indicates the null effect (HR/OR = 1.0).

Taken together, higher CALLY values were associated with more favorable outcomes, whereas higher CAR/hsCAR, NPAR, and LAR were associated with adverse outcomes in CAD populations (Figure 6).

### 3.3. Discriminative Performance and Clinical Utility of Albumin-Based Biomarkers

For long-term MACEs, bivariate hierarchical analysis showed that CAR achieved the highest summary AUC (0.76), with relatively balanced sensitivity (0.70) and specificity (0.70). NPAR also showed good discriminatory performance (AUC = 0.74) and was characterized by higher pooled specificity (0.80), whereas CALLY showed a more moderate discriminative profile (AUC = 0.69).

Fagan’s nomogram analysis based on a pre-test probability of 20.0% further illustrated differences in potential clinical utility. A positive NPAR result increased the post-test probability of MACEs to 46.7% (PLR = 3.50), representing the strongest upward shift in risk. By contrast, CAR showed a more balanced risk reclassification profile, increasing the post-test probability to 37.1% after a positive result and reducing it to 9.6% after a negative result (NLR = 0.42).

For all-cause and in-hospital mortality, both CAR and CALLY achieved a summary AUC of 0.79, although their performance profiles differed: CAR showed higher sensitivity (0.78), whereas CALLY showed higher specificity (0.82). LAR also showed good prognostic performance for mortality, with a summary AUC of 0.74, a sensitivity of 0.68, and a specificity of 0.73 (Figure S5). Using a pre-test probability of 10.0%, Fagan’s nomogram analysis showed that CALLY produced the largest post-test increase after a positive result (PLR = 3.56; post-test probability = 28.3%), whereas CAR yielded the lowest post-test probability after a negative result (NLR = 0.34; post-test probability = 3.6%) (Figure S6).

**Figure 7:**
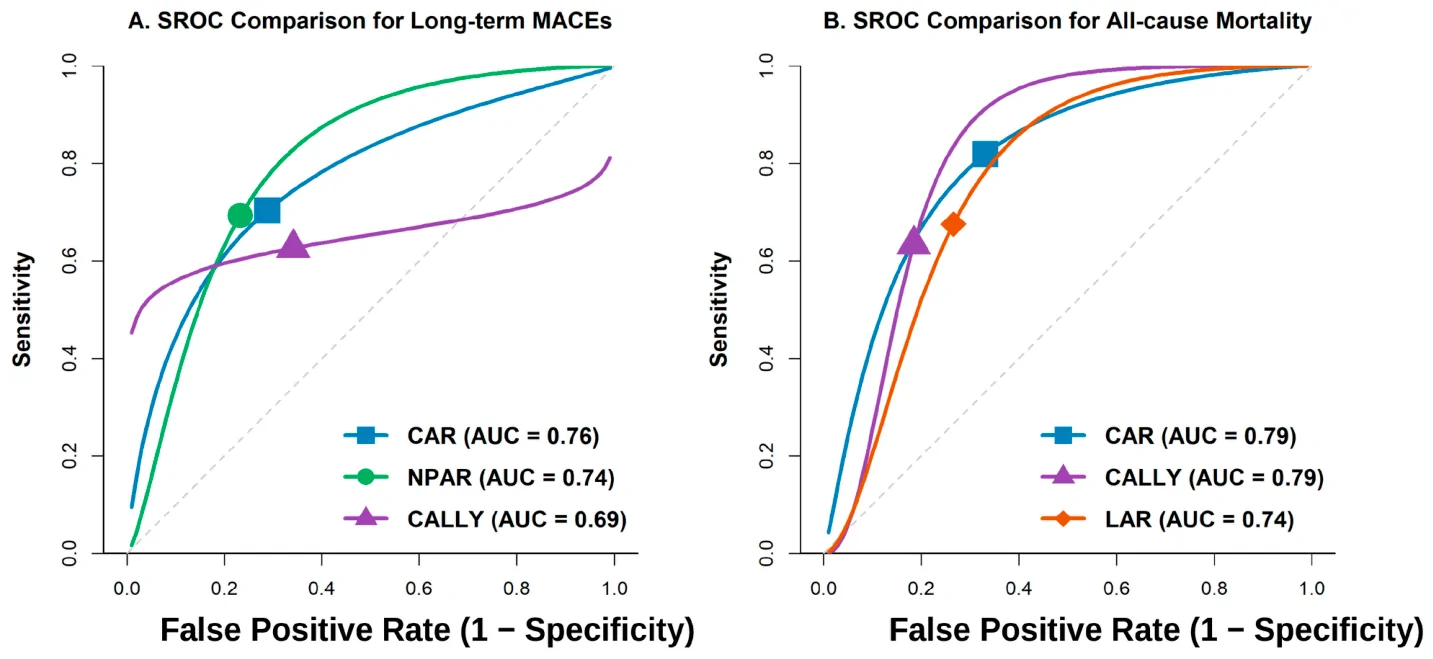
Summary receiver operating characteristic (SROC) curves for discriminative performance of selected albumin-based composite biomarkers. Abbreviations: CAR, C-reactive protein-to-albumin ratio; NPAR, neutrophil percentage-to-albumin ratio; LAR, lactate-to-albumin ratio; CALLY, C-reactive protein–albumin–lymphocyte index; SROC, summary receiver operating characteristic; AUC, area under the curve; MACE, major adverse cardiovascular events.

Head-to-head comparison suggested an endpoint-specific performance pattern (Figure 7 and Table S4). CAR showed relatively balanced discrimination across both MACEs and mortality, NPAR showed the strongest positive risk shift for long-term MACEs, and CALLY and LAR showed stronger discrimination for mortality.

### 3.4. Sensitivity Analysis and Publication Bias

Leave-one-out sensitivity analyses were performed for pooled outcomes including four or more studies to assess result stability and explore potential sources of heterogeneity (Figure S7).

Overall, the pooled associations for CAR/hsCAR and MACE, NPAR and mortality, and LAR and mortality were stable. For CAR/hsCAR and MACE, sequential omission of individual studies did not materially alter the significance of the pooled estimate. Notably, exclusion of Huang et al. [24] reduced heterogeneity from 59.9% to 0.0%, while the association remained significant (HR, 1.32; 95% CI, 1.24–1.40). Similarly, the associations of NPAR and LAR with mortality remained significant in all leave-one-out analyses, with all recalculated lower confidence bounds remaining above 1.0. By contrast, pooled estimates based on fewer studies, particularly CALLY and mortality and CAR and mortality, were more sensitive to omission of individual cohorts. Exclusion of certain studies, including Pan [28] or Arslan [23], resulted in confidence intervals crossing unity. However, the direction of effect was unchanged, with recalculated point estimates remaining protective for CALLY (HR range, 0.40–0.58) and adverse for CAR (OR range, 1.33–2.02). These findings suggest that the overall effect direction was preserved, although the precision of the pooled estimates was more limited in analyses with smaller numbers of studies. Formal publication bias analyses were not performed. All pooled outcomes included fewer than 10 studies, under which funnel plots and asymmetry tests are generally considered underpowered and potentially unreliable.

## 4. Discussion

In this systematic review and meta-analysis, albumin-based biomarkers showed distinct but clinically meaningful associations with adverse outcomes across CAD populations. Two broad patterns were evident. Higher CALLY values were generally associated with more favorable outcomes, whereas elevated CAR/hsCAR, NPAR, and LAR were associated with a worse prognosis. NPAR showed the most consistent association with mortality, with little between-study heterogeneity, while LAR yielded the largest pooled mortality effect estimate. CAR/hsCAR, evaluated exclusively in ACS-related cohorts, was associated with both mortality and MACE, supporting a broader prognostic role within the ACS setting. By contrast, the pooled estimates for CALLY and NPAR-related MACE were less stable despite consistent directional trends, suggesting that the strength and maturity of evidence varied across biomarkers and endpoints. Discriminative analyses also suggested differences in potential clinical utility, with CAR showing relatively balanced performance across endpoints, NPAR showing a stronger rule-in profile for long-term MACEs, and CALLY and LAR appearing more relevant to mortality prediction.

These differing prognostic patterns are biologically plausible and may reflect the different dimensions of risk captured by these albumin-based biomarkers. Although all four markers share albumin as a common component, albumin is paired with biologically distinct variables that map onto inflammation, innate and adaptive immunity, and metabolic stress [48]. CAR/hsCAR combines albumin with C-reactive protein, a prototypical positive acute-phase reactant, and therefore primarily reflects inflammatory burden in the setting of a reduced negative acute-phase protein response (hypoalbuminemia) [49,50]. NPAR integrates albumin with the neutrophil percentage, which may more directly capture innate immune activation and systemic inflammatory imbalance; similar neutrophil-based indices (e.g., neutrophil-to-lymphocyte ratio, systemic-inflammation index) consistently track adverse outcomes in cardiovascular and other high-risk settings, supporting the particularly stable association between NPAR and mortality [51,52]. LAR, in contrast, combines albumin with lactate and is therefore more closely related to metabolic stress, tissue hypoperfusion, and acute physiological instability, pathways strongly linked to short-term fatal outcomes in acute cardiovascular and critical-illness contexts [48,49]. By comparison, the CALLY index incorporates CRP, albumin, and lymphocyte count, and may therefore reflect not only inflammation and nutritional reserve but also immune competence, akin to other lymphocyte-based immune–inflammatory scores used in prognostication [53]. In this framework, a higher CALLY value would be expected to indicate a more preserved systemic state-lower inflammatory drive, better nutritional/physiologic reserve, and intact adaptive immunity-and, accordingly, a lower risk of adverse outcomes.

This biological heterogeneity is also relevant when the present findings are considered alongside the broader literature on composite inflammatory biomarkers in coronary disease. Most previous studies in ACS and related coronary populations have focused on blood cell–based indices such as the neutrophil-to-lymphocyte ratio (NLR), platelet-to-lymphocyte ratio (PLR), monocyte-to-lymphocyte ratio (MLR), and the systemic immune-inflammation or response indices (SII/SIRI), which have repeatedly been associated with coronary disease severity and adverse cardiovascular outcomes [54,55]. These markers are generally regarded as indicators of systemic inflammatory activation and immune imbalance in atherosclerosis and ACS. By contrast, the biomarkers examined in the present study share albumin as a common component and may therefore capture not only inflammation, but also nutritional reserve and, in the case of LAR, metabolic or hemodynamic stress [56,57]. This difference in construct may partly explain why the present analysis identified distinct prognostic patterns across markers rather than a single uniform signal. The current study extends this literature by evaluating CALLY, CAR/hsCAR, NPAR, and LAR within a single framework and by showing that these indices differ not only in the direction of association, but also in endpoint-specific stability and potential clinical relevance. At the same time, the review was intentionally selective rather than exhaustive. Biomarkers supported by only sparse cardiovascular evidence were not prioritized for quantitative synthesis, whereas markers such as the fibrinogen-to-albumin ratio, which have already been reviewed in related coronary settings, were not re-synthesized here [11].

An important consideration is that these albumin-based biomarkers are not specific to coronary pathology. Albumin concentrations may be affected by hepatic synthetic dysfunction, renal impairment or protein loss, malnutrition, systemic inflammation, and active infection, while the other components of these indices may also vary with acute illness and treatment. Concomitant medications may further modify biomarker levels; statins, for example, can influence inflammatory markers such as CRP/hs-CRP and thereby potentially affect CAR/hsCAR. Therefore, these indices should be interpreted as integrative markers of systemic physiological stress rather than direct measures of coronary disease burden. Although we preferentially extracted the most fully adjusted estimates, adjustment for these potential confounders was not uniform across the included studies.

The variability in association strength and stability across biomarkers likely reflects both endpoint-related and population-related heterogeneity. In particular, ACS and chronic CAD represent different clinical settings: acute inflammatory activation, plaque instability, ischemic stress, and hemodynamic disturbance are generally more prominent in ACS, whereas chronic CAD reflects a relatively more stable atherosclerotic state. These differences may influence the levels and prognostic relevance of albumin-based biomarkers. Associations with mortality were more consistent than those with MACE, particularly for NPAR and LAR. This is not unexpected, as mortality is a relatively hard endpoint with less variation in definition across studies, whereas MACE/MACCE are composite outcomes that differ in their components, follow-up windows, and adjudication. Such variation may partly explain why some biomarkers showed stable associations with mortality but less robust associations with broader cardiovascular event outcomes. A second source of heterogeneity was population composition. Although most included studies were based on ACS-spectrum cohorts, several enrolled broader CAD populations. To address this, additional subgroup analyses were performed where data permitted. The association between NPAR and mortality was directionally consistent across both ACS-related and broader CAD cohorts, with no significant subgroup difference, and the association between LAR and mortality remained evident after restriction to ACS-related studies. These findings suggest that the main mortality signals were not solely driven by differences in endpoint definition or population mix, although caution is still warranted for results supported by fewer studies, particularly those involving CALLY and NPAR-related MACE.

From a clinical standpoint, these biomarkers are appealing because they are derived from routine laboratory tests and can be obtained early without additional cost or procedural burden. This makes them potentially useful as adjunctive tools for risk stratification, especially in settings where timely assessment is needed. The present results also suggest that their clinical roles may differ. CAR showed relatively balanced performance across both mortality and MACE, whereas NPAR appeared to carry a stronger signal for long-term cardiovascular event risk. CALLY and LAR, in contrast, seemed more informative for mortality. These differences suggest that the biomarkers may be better suited to different clinical questions, rather than serving interchangeable roles. However, the current evidence does not support the use of any single biomarker as a stand-alone prognostic tool. Their practical value may lie in complementing established clinical assessment and conventional risk models by providing information on systemic inflammatory, nutritional, immune, or metabolic status. Importantly, most included studies did not formally assess whether these biomarkers improved discrimination or risk prediction beyond established models. Whether they provide incremental prognostic value beyond established risk models requires prospective validation.

### Limitations

Several limitations should be acknowledged. First, most included studies were retrospective, limiting causal inference and leaving room for residual confounding. In particular, hepatic dysfunction, renal impairment, malnutrition, active infection, and concomitant medication use may influence albumin or other components of these indices independently of cardiovascular status, but these factors were not uniformly reported or adjusted for across studies. Second, biomarker definitions and analytic strategies varied across studies, including differences in cutoff selection, categorization, and treatment as continuous or grouped variables. Third, the study populations were not entirely homogeneous. Most evidence came from ACS-related cohorts, whereas some studies included broader or more stable CAD populations. Given the clinical and pathophysiological differences between acute and chronic coronary disease, pooled estimates across these settings should be interpreted cautiously, and extrapolation to stable or non-ACS populations remains uncertain. Fourth, outcome definitions—particularly for MACE/MACCE—were not fully consistent across studies, likely contributing to heterogeneity. Fifth, several pooled analyses were based on a limited number of studies, and some findings, especially those involving CALLY and NPAR-related MACE, were less stable in the sensitivity analyses. Finally, the predominance of studies from China and Turkey may limit generalizability to other populations. Future work should emphasize prospective validation, more standardized biomarker modeling, clearer endpoint definitions, and assessment of whether these markers add prognostic value beyond established risk scores in specific coronary settings.

## 5. Conclusions

Albumin-based biomarkers were associated with adverse outcomes across coronary disease populations, particularly in ACS-related settings. The direction and strength of these associations varied across biomarkers and endpoints, with more consistent evidence observed for NPAR-related mortality, LAR-related mortality, and CAR-associated adverse outcomes in ACS. These biomarkers may provide complementary prognostic information but should not replace established clinical and prognostic assessment. Further prospective studies are needed to determine their incremental clinical value.

## Figures and Tables

**Table 1:** Characteristics of included studies.

Study	Country	Design	No. of Patients	Population	Age, Years	Male, %	Cutoff/ Grouping	Biomarker	Follow-Up, Months	Outcome(s)
Çınar 2019 [14]	Turkey	Retrospective	2243	STEMI-PCI	57 ± 12	81.5	Tertiles	CAR	34.0 ± 15.0	All-cause mortality
Wang 2019 [15]	China	Prospective	652	ACS	66.49 ± 11.07	45.4	Dichotomized (0.114)	CAR	5.6 (0.5–6.0)	In-hospital and short-term MACE
Cui 2019 [34]	China	Retrospective	1024	STEMI	64.07 ± 12.65	73.5	Tertiles: Q1 < 1.75; Q2 1.75–2.05; Q3 > 2.05	NPAR	In-hospital	In-hospital mortality, MACE; cardiac death
Sun 2020 [35]	China	Retrospective	3106	CAD	72.5 (63.6–81.0)	63.6	Tertiles: Q1 < 22.1; Q2 22.1–27.9; Q3 ≥ 27.9	NPAR	12.0	Mortality (multiple time points)
Acet 2021 [16]	Turkey	Retrospective	539	STEMI-PCI	62.3 + 13.8	69.2	Dichotomized (0.395)	CAR	6.0	MACE
SÖĞÜT 2021 [17]	Turkey	Retrospective	116	STEMI-PCI	56.47 ± 11.42	86.2	Dichotomized (cutoff NR)	CAR	NR	Mortality
Zhang 2021 [18]	China	Retrospective	1722	ACS	59.34 ± 11	76.2	Dichotomized (cutoff NR)	hsCAR	NR	Mortality
Lin 2022 [36]	China	Retrospective	798	AMI	69.83 (57.0–80.00)	63.2	Tertiles: Q1 < 21.58; Q2 21.58–26.77; Q3 ≥ 26.77	NPAR	12.0	Mortality (multiple time points)
Zhu 2022 [42]	China	Retrospective	843	AMI	67.73 ± 12.76	65.1	Tertiles: Q1 ≤ 0.47; Q2 0.47–0.97; Q3 > 0.97	LAR	1.0	30-day all-cause mortality
Birda 2023 [19]	Turkey	Retrospective	1689	ACS	56.6 ± 12.2	80.5	Dichotomized (cutoff NR)	CAR	38.9 ± 10.3	Long-term mortality
Li 2023 [20]	China	Retrospective	429	NSTEMI	59.28 (51.0–68.0)	74.8	Dichotomized (cutoff NR)	hsCAR	6.0	MACE; coronary artery severity
Chen 2023 [43]	China	Retrospective	2626	MI	67.48 (67.02–67.95)	66.0	Dichotomized (0.671)	LAR	NR	In-hospital mortality
Wang 2023 [44]	China	Retrospective	1134	AMI	68.87	67.9	Tertiles: Q1 < 0.4063; Q2 0.4063–0.6667; Q3 > 0.6667	LAR	NR	All-cause mortality
Huang 2024 [21]	China	Retrospective	699	STEMI	66.0 (58.0, 73.0)	74.1	Dichotomized (0.15)	hsCAR	32.9	MACE
Menekşe 2024 [22]	Turkey	Retrospective	308	NSTEMI	64.05 ± 11.62	64.6	Dichotomized (0.864)	CAR	In-hospital	In-hospital mortality
Karaca 2024 [37]	Turkey	Retrospective	464	NSTEMI	59.2 ± 11.6	79.1	Dichotomized (17.6)	NPAR/CAR	12.0	MACCE
Pan 2024 [28]	China	Retrospective	3799	CAD-PCI	61.78 ± 11.99	73.0	Quartiles: Q1 ≤ 0.69; Q2 0.69–2.44; Q3 2.44–9.52; Q4 > 9.52	CALLY	24.0	All-cause mortality; Cardiac mortality; MACE
Ji 2024 [27]	China	Retrospective	438	STEMI-PCI	61.0 (0.67)	80.1	Dichotomized (0.758)	CALLY	30.0	MACE
Jin 2024 [46]	China	Retrospective	989	AMI	68.28 ± 12.92	44.1	NR	LAR	0.9	28-day all-cause mortality
Chen 2024 [45]	China	Retrospective	2816	AMI	65.04 (64.57–65.53)	62.7	Dichotomized (0.709)	LAR	NR	In-hospital mortality
Liu 2024 [47]	China	Retrospective	1902	CHD	71.62 ± 13.26	39.2	Quartiles: Q1 0.29 ± 0.06; Q2 0.46 ± 0.05; Q3 0.71 ± 0.10; Q4 1.86 ± 1.13	LAR	0.9	28-day mortality
Arslan 2025 [23]	Turkey	Retrospective	420	ACS	63.4 ± 11.8	68.1	Dichotomized (2.8)	CAR	In-hospital	In-hospital mortality
Huang 2025 [24]	China	Retrospective	1177	STEMI-PCI	66 (57, 72)	77.0	Dichotomized (0.18)	CAR	15.1	MACE
Oflar 2025 [25]	Turkey	Retrospective	1142	NSTEMI-PCI	61.9 ± 12.5	74.3	Dichotomized (cutoff NR)	CAR	12.0	MACCE
Cao 2025 [38]	China	Retrospective	1759	MI	70.67 (62.01, 79.60)	65.0	Tertiles: Q1 18.85 (17.26–20.22); Q2 23.82 (22.60–25.30); Q3 31.25 (28.92–35.50)	NPAR	12.0	30-day and 360-day all-cause mortality
Li 2025 [39]	China	Retrospective	3163	CAD	65.04 ± 0.27	56.2	Tertiles: Q1 < 13.5; Q2 13.5–15.8; Q3 ≥ 15.8	NPAR	76.0	Cardiovascular and all-cause mortality
Yan 2025 [40]	China	Retrospective	935	CAD	70.00 (17.00)	67.6	Tertiles: Q1 ≤ 13.49; Q2 13.49–15.83; Q3 > 15.83	NPAR	110.0	Cardiovascular and all-cause mortality
Güven 2025 [30]	Turkey	Retrospective	505	ACS-PCI	59 (52–68)	76.6	Dichotomized (0.7)	CALLY	40.0	All-cause mortality
Han 2025 [31]	China	Retrospective	2183	CAD	63.38 (14.04)	53.7	Quartiles (Q1–Q4)	CALLY	122.0	All-cause and cardiovascular mortality
Wen 2025 [32]	China	Retrospective	4313	CAD	67.10 (12.95)	52.2	NR	CALLY	NR	All-cause and cardiovascular mortality
Demir 2025 [29]	Turkey	Retrospective	1133	STEMI-PCI	56 ± 12	82.1	Dichotomized (3.03)	CALLY	In-hospital	In-hospital mortality
Li 2026 [26]	China	Retrospective	752	STEMI-PCI	62 ± 11	75.5	Dichotomized (0.127)	hsCAR	52.0	MACE; All-cause mortality
Wang 2026 [41]	China	Retrospective	1524	ACS-PCI	58.91 ± 10.32	74.9	Dichotomized (17.326)	NPAR	12.0	MACE
Xu 2026 [33]	China	Retrospective	421	STEMI-PCI	60.64 (49–74)	76.5	Dichotomized (0.77)	CALLY/CAR	24.0	MACE

Abbreviations: ACS, acute coronary syndrome; AMI, acute myocardial infarction; CAD, coronary artery disease; CAG, coronary angiography; CAR, C-reactive protein-to-albumin ratio; CALLY, C-reactive protein–albumin–lymphocyte index; CHD, coronary heart disease; CVD, cardiovascular disease; hsCAR, high-sensitivity C-reactive protein-to-albumin ratio; IQR, interquartile range; LAR, lactate-to-albumin ratio; MACE, major adverse cardiovascular events; MACCE, major adverse cardiac and cerebrovascular events; MI, myocardial infarction; NPAR, neutrophil percentage-to-albumin ratio; NR, not reported; NSTEMI, non-ST-segment elevation myocardial infarction; PCI, percutaneous coronary intervention; STEMI, ST-segment elevation myocardial infarction.

## Data Availability

Data sharing is not applicable to this article as no datasets were generated or analyzed in the current study.

## Supplementary Materials

The following supporting information can be downloaded at https://www.mdpi.com/article/10.3390/jcm15176868/s1. Table S1. PRISMA 2020 Checklist; Table S2. Search Strategy; Table S3. Newcastle–Ottawa Scale assessment of included studies; Table S4. Diagnostic and Prognostic Performance; Figure S1. Subgroup analysis of CAR/hsCAR with MACE by biomarker subtype; Figure S2. Subgroup analysis of NPAR and mortality by follow-up duration; Figure S3. Subgroup analysis of NPAR and mortality by population type; Figure S4. Association between LAR and mortality in ACS cohorts; Figure S5. Discriminative performance of albumin derived indices for adverse outcomes in CVD; Figure S6. Clinical utility of albumin-derived indices for adverse outcome prediction; Figure S7. Leave-one-out sensitivity analyses.
