# Supplementary material for: Albumin-Based Biomarkers and Adverse Outcomes in Coronary Artery Disease: A Systematic Review and Meta-Analysis
Source: J Clin Med. 2026 Sep 4;15(17):6868. doi: 10.3390/jcm15176868 (PMC13566714; doi:10.3390/jcm15176868)

Supplementary Materials

Title: Albumin-Based Biomarkers and Adverse Outcomes in Coronary Artery Disease: A Systematic Review and Meta-Analysis

Table of Contents

- Supplementary Tables
  - - Table S1. PRISMA 2020 Checklist .... Page S2-5
  - - Table S2. Search Strategy .... Page S6-7
  - - Table S3. Newcastle–Ottawa Scale assessment of included studies .... Page S8-9
  - - Table S4. Diagnostic and Prognostic Performance .... Page S10
- Supplementary Figures
  - - Figure S1. Subgroup analysis of CAR/hsCAR with MACE by biomarker subtype. .... Page S11
  - - Figure S2. Subgroup analysis of NPAR and mortality by follow-up duration .... Page S12
  - - Figure S3. Subgroup analysis of NPAR and mortality by population type............................. Page S13
  - - Figure S4. Association between LAR and mortality in ACS cohorts............................. Page S14
  - - Figure S5. Discriminative performance of albumin-derived indices for adverse outcomes in CVD............................. Page S15-16
  - - Figure S6. Clinical utility of albumin-derived indices for adverse outcome prediction............................. Page S17
  - - Figure S7. Leave-one-out sensitivity analyses............................. Page S18

Table S1: PRISMA 2020 Checklist

Section and Topic	Item #	Checklist item	Location where item is reported
**TITLE**			
Title	1	Identify the report as a systematic review.	1
**ABSTRACT**			
Abstract	2	See the PRISMA 2020 for Abstracts checklist.	1
**INTRODUCTION**			
Rationale	3	Describe the rationale for the review in the context of existing knowledge.	2
Objectives	4	Provide an explicit statement of the objective(s) or question(s) the review addresses.	2,3
**METHODS**			
Eligibility criteria	5	Specify the inclusion and exclusion criteria for the review and how studies were grouped for the syntheses.	4
Information sources	6	Specify all databases, registers, websites, organisations, reference lists and other sources searched or consulted to identify studies. Specify the date when each source was last searched or consulted.	3
Search strategy	7	Present the full search strategies for all databases, registers and websites, including any filters and limits used.	3
Selection process	8	Specify the methods used to decide whether a study met the inclusion criteria of the review, including how many reviewers screened each record and each report retrieved, whether they worked independently, and if applicable, details of automation tools used in the process.	4
Data collection process	9	Specify the methods used to collect data from reports, including how many reviewers collected data from each report, whether they worked independently, any processes for obtaining or confirming data from study investigators, and if applicable, details of automation tools used in the process.	4
Data items	10a	List and define all outcomes for which data were sought. Specify whether all results that were compatible with each outcome	4

Section and Topic	Item #	Checklist item	Location where item is reported
		domain in each study were sought (e.g. for all measures, time points, analyses), and if not, the methods used to decide which results to collect.	
	10b	List and define all other variables for which data were sought (e.g. participant and intervention characteristics, funding sources). Describe any assumptions made about any missing or unclear information.	4
Study risk of bias assessment	11	Specify the methods used to assess risk of bias in the included studies, including details of the tool(s) used, how many reviewers assessed each study and whether they worked independently, and if applicable, details of automation tools used in the process.	4,5
Effect measures	12	Specify for each outcome the effect measure(s) (e.g. risk ratio, mean difference) used in the synthesis or presentation of results.	5
Synthesis methods	13a	Describe the processes used to decide which studies were eligible for each synthesis (e.g. tabulating the study intervention characteristics and comparing against the planned groups for each synthesis (item #5)).	5
	13b	Describe any methods required to prepare the data for presentation or synthesis, such as handling of missing summary statistics, or data conversions.	5
	13c	Describe any methods used to tabulate or visually display results of individual studies and syntheses.	5
	13d	Describe any methods used to synthesize results and provide a rationale for the choice(s). If meta-analysis was performed, describe the model(s), method(s) to identify the presence and extent of statistical heterogeneity, and software package(s) used.	5
	13e	Describe any methods used to explore possible causes of heterogeneity among study results (e.g. subgroup analysis, meta-regression).	5
	13f	Describe any sensitivity analyses conducted to assess robustness of the synthesized results.	5
Reporting bias assessment	14	Describe any methods used to assess risk of bias due to missing results in a synthesis (arising from reporting biases).	5

Section and Topic	Item #	Checklist item	Location where item is reported
Certainty assessment	15	Describe any methods used to assess certainty (or confidence) in the body of evidence for an outcome.	5
**RESULTS**			
Study selection	16a	Describe the results of the search and selection process, from the number of records identified in the search to the number of studies included in the review, ideally using a flow diagram.	6
	16b	Cite studies that might appear to meet the inclusion criteria, but which were excluded, and explain why they were excluded.	6
Study characteristics	17	Cite each included study and present its characteristics.	6
Risk of bias in studies	18	Present assessments of risk of bias for each included study.	8,9
Results of individual studies	19	For all outcomes, present, for each study: (a) summary statistics for each group (where appropriate) and (b) an effect estimate and its precision (e.g. confidence/credible interval), ideally using structured tables or plots.	7,8
Results of syntheses	20a	For each synthesis, briefly summarise the characteristics and risk of bias among contributing studies.	6
	20b	Present results of all statistical syntheses conducted. If meta-analysis was done, present for each the summary estimate and its precision (e.g. confidence/credible interval) and measures of statistical heterogeneity. If comparing groups, describe the direction of the effect.	7
	20c	Present results of all investigations of possible causes of heterogeneity among study results.	7,8
	20d	Present results of all sensitivity analyses conducted to assess the robustness of the synthesized results.	8,9
Reporting biases	21	Present assessments of risk of bias due to missing results (arising from reporting biases) for each synthesis assessed.	9

Section and Topic	Item #	Checklist item	Location where item is reported
Certainty of evidence	22	Present assessments of certainty (or confidence) in the body of evidence for each outcome assessed.	7,8
**DISCUSSION**			
Discussion	23a	Provide a general interpretation of the results in the context of other evidence.	9,10,11
	23b	Discuss any limitations of the evidence included in the review.	11
	23c	Discuss any limitations of the review processes used.	11
	23d	Discuss implications of the results for practice, policy, and future research.	11,12
**OTHER INFORMATION**			
Registration and protocol	24a	Provide registration information for the review, including register name and registration number, or state that the review was not registered.	/
	24b	Indicate where the review protocol can be accessed, or state that a protocol was not prepared.	/
	24c	Describe and explain any amendments to information provided at registration or in the protocol.	/
Support	25	Describe sources of financial or non-financial support for the review, and the role of the funders or sponsors in the review.	
Competing interests	26	Declare any competing interests of review authors.	13
Availability of data, code and other materials	27	Report which of the following are publicly available and where they can be found: template data collection forms; data extracted from included studies; data used for all analyses; analytic code; any other materials used in the review.	/

Table S2. Search strategies for Each Database

Database	Search Strategy
PubMed (MEDLINE)<br>（n=198）	(("Acute Coronary Syndrome"[Mesh] OR "Myocardial Infarction"[Mesh] OR "Coronary Artery Disease"[Mesh] OR "Percutaneous Coronary Intervention"[Mesh] OR "acute coronary syndrome"[tiab] OR ACS[tiab] OR "myocardial infarction"[tiab] OR AMI[tiab] OR STEMI[tiab] OR NSTEMI[tiab] OR "coronary artery disease"[tiab] OR CAD[tiab] OR "coronary heart disease"[tiab] OR CHD[tiab] OR "cardiovascular disease"[tiab] OR CVD[tiab] OR "percutaneous coronary intervention"[tiab] OR PCI[tiab]) AND ("C-reactive protein-to-albumin ratio"[tiab] OR "CRP-to-albumin ratio"[tiab] OR CAR[tiab] OR hsCAR[tiab] OR "neutrophil percentage-to-albumin ratio"[tiab] OR NPAR[tiab] OR "lactate-to-albumin ratio"[tiab] OR LAR[tiab] OR "C-reactive protein-albumin-lymphocyte index"[tiab] OR CALLY[tiab]) AND ("Mortality"[Mesh] OR "Survival"[Mesh] OR mortality[tiab] OR death[tiab] OR survival[tiab] OR prognosis[tiab] OR prognostic[tiab] OR outcome[tiab] OR outcomes[tiab] OR "major adverse cardiovascular event*"[tiab] OR MACE[tiab] OR MACCE[tiab] OR "major adverse cardiac and cerebrovascular event*"[tiab]) AND ("Cohort Studies"[Mesh] OR "Case-Control Studies"[Mesh] OR "Observational Study"[Publication Type] OR cohort[tiab] OR cohorts[tiab] OR prospective[tiab] OR retrospective[tiab] OR observational[tiab])) NOT (animals[mh] NOT humans[mh])
Embase (Elsevier)<br>(n=420)	1. Coronary disease / ACS concept ('acute coronary syndrome'/exp OR 'myocardial infarction'/exp OR 'coronary artery disease'/exp OR 'percutaneous coronary intervention'/exp OR 'acute coronary syndrome':ti,ab OR ACS:ti,ab OR 'myocardial infarction':ti,ab OR 'acute myocardial infarction':ti,ab OR AMI:ti,ab OR STEMI:ti,ab NSTEMI:ti,ab OR 'coronary artery disease':ti,ab OR CAD:ti,ab OR 'coronary heart disease':ti,ab OR CHD:ti,ab OR 'cardiovascular disease':ti,ab OR CVD:ti,ab OR 'percutaneous coronary intervention':ti,ab OR PCI:ti,ab)<br>2. Selected albumin-based composite biomarkers concept ('c reactive protein to albumin ratio':ti,ab OR 'crp to albumin ratio':ti,ab OR CAR:ti,ab OR hsCAR:ti,ab OR 'neutrophil percentage to albumin ratio':ti,ab OR NPAR:ti,ab OR 'lactate to albumin ratio':ti,ab OR LAR:ti,ab OR 'c reactive protein albumin lymphocyte index':ti,ab OR 'crp albumin lymphocyte index':ti,ab OR CALLY:ti,ab)<br>3. Prognosis / adverse outcomes concept ('mortality'/exp OR 'survival'/exp OR mortality:ti,ab OR death:ti,ab OR survival:ti,ab OR prognosis:ti,ab OR prognostic:ti,ab OR outcome:ti,ab OR outcomes:ti,ab OR 'major adverse cardiovascular event':ti,ab OR MACE:ti,ab OR MACCE:ti,ab OR 'major adverse cardiac and cerebrovascular event':ti,ab)<br>3. Study design filter ('cohort analysis'/exp OR 'case control study'/exp OR 'prospective study'/exp OR 'retrospective study'/exp OR 'observational study'/exp OR cohort:ti,ab OR cohorts:ti,ab OR prospective:ti,ab OR retrospective:ti,ab OR observational:ti,ab)<br>4. Combined search 1 AND 2 AND 3 AND 4<br>5.Exclusion (exp animal/ OR nonhuman/) NOT exp human/
Web of Science Core	**#1 Coronary disease / ACS topic**

Collection<br>(n=538)	TS=("acute coronary syndrome" OR ACS OR "myocardial infarction" OR "acute myocardial infarction" OR AMI<br>OR STEMI OR NSTEMI OR "coronary artery disease" OR CAD OR "coronary heart disease" OR CHD<br>OR "cardiovascular disease" OR CVD OR "percutaneous coronary intervention" OR PCI)<br>**#2 Selected albumin-based composite biomarkers topic**<br>TS=("C-reactive protein-to-albumin ratio" OR "C reactive protein-to-albumin ratio" OR "CRP-to-albumin ratio" OR CAR OR hsCAR<br>OR "neutrophil percentage-to-albumin ratio" OR "neutrophil percentage to albumin ratio" OR NPAR<br>OR "lactate-to-albumin ratio" OR "lactate to albumin ratio" OR LAR<br>OR "C-reactive protein-albumin-lymphocyte index" OR "CRP-albumin-lymphocyte index" OR CALLY)<br>**#3 Prognosis / mortality topic**<br>TS=(mortality OR death OR survival OR prognosis OR prognostic OR outcome OR outcomes OR "major adverse cardiovascular event*" OR MACE OR MACCE<br>OR "major adverse cardiac and cerebrovascular event*")<br>**#4 Study design topic**<br>TS=(cohort OR cohorts OR prospective OR retrospective OR observational)<br>**#5 Combined search**<br>#1 AND #2 AND #3 AND #4

Table S3. Newcastle–Ottawa Scale assessment of included studies

Study (Year)	Selection (Max 4)	Comparability (Max 2)	Outcome (Max 3)	Total Score (Max 9)
Çınar 2019	4	2	2	8
Wang 2019	4	2	2	8
Cui 2019	3	1	2	6
Sun 2020	4	2	2	8
Acet 2021	3	2	2	7
Söğüt 2021	3	1	2	6
Zhang 2021	4	1	2	7
Lin 2022	4	2	2	8
Zhu 2022	3	2	2	7
Birda 2023	4	2	2	8
Li 2023	3	2	2	7
Chen 2023	4	1	2	7
Wang 2023	4	2	2	8
Huang 2024	3	2	2	7
Menekşe 2024	3	1	2	6
Karaca 2024	4	2	2	8
Pan 2024	4	2	3	9
Ji 2024	3	2	2	7
Jin 2024	3	2	2	7
Chen 2024	4	1	2	7
Liu 2024	4	2	2	8
Arslan 2025	3	1	2	6
Huang 2025	4	2	2	8

**Oflar 2025**	**3**	**2**	**2**	**7**
**Cao 2025**	**4**	**2**	**2**	**8**
**Li 2025**	**4**	**2**	**3**	**9**
**Yan 2025**	**4**	**2**	**2**	**8**
**Güven 2025**	**3**	**2**	**2**	**7**
**Han 2025**	**4**	**2**	**3**	**9**
**Wen 2025**	**4**	**2**	**2**	**8**
**Demir 2025**	**3**	**1**	**2**	**6**
**Li 2026**	**3**	**2**	**2**	**7**
**Wang 2026**	**4**	**2**	**2**	**8**
**Xu 2026**	**3**	**2**	**2**	**7**

Table S4: Comprehensive Summary of Diagnostic and Prognostic Performance of Immuno-nutritional Biomarkers in CAD Patients

Biomarker	Clinical Endpoint	Studies (n)	Total Patients (N)	Pooled Sensitivity	Pooled Specificity	PLR	NLR	Summary AUC	Post-test Prob. (+)*	Post-test Prob. (-)*
**CAR**	Long-term MACEs	3	2,368	0.70	0.70	2.36	0.42	**0.76**	37.1%	9.6%
	All-cause Mortality	3	2,782	0.78	0.65	2.23	0.34	**0.79**	19.9%	3.6%
**NPAR**	Long-term MACEs	2	1,988	0.70	0.80	**3.50**	0.38	**0.74**	**46.7%**	8.6%
**CALLY**	Long-term MACEs	2	836	0.62	0.66	1.82	0.58	**0.69**	31.3%	12.7%
	All-cause Mortality	3	2,053	0.64	**0.82**	**3.56**	0.44	**0.79**	**28.3%**	4.7%
**LAR**	All-cause Mortality	3	7,344	0.68	0.73	2.52	0.44	**0.74**	21.8%	4.6%

Abbreviations: CAR, C-reactive protein to albumin ratio; **NPAR**, Neutrophil percentage-to-albumin ratio; **CALLY**, CRP-albumin-lymphocyte index; **LAR**, Lactic acid to albumin ratio; **MACEs**, Major adverse cardiovascular events; **PLR**, Positive likelihood ratio; **NLR**, Negative likelihood ratio; **AUC**, Area under the summary receiver operating characteristic curve.

***Post-test Probabilities** were calculated using Fagan's nomogram based on pre-test probabilities of 20.0% for MACEs and 10.0% for Mortality. These values conceptually illustrate the clinical risk reclassification utility (rule-in and rule-out capacities) of each biomarker in a real-world clinical setting.

Figure S1. Subgroup analysis of the association between CAR/hsCAR and major adverse cardiovascular events (MACE) according to biomarker subtype.

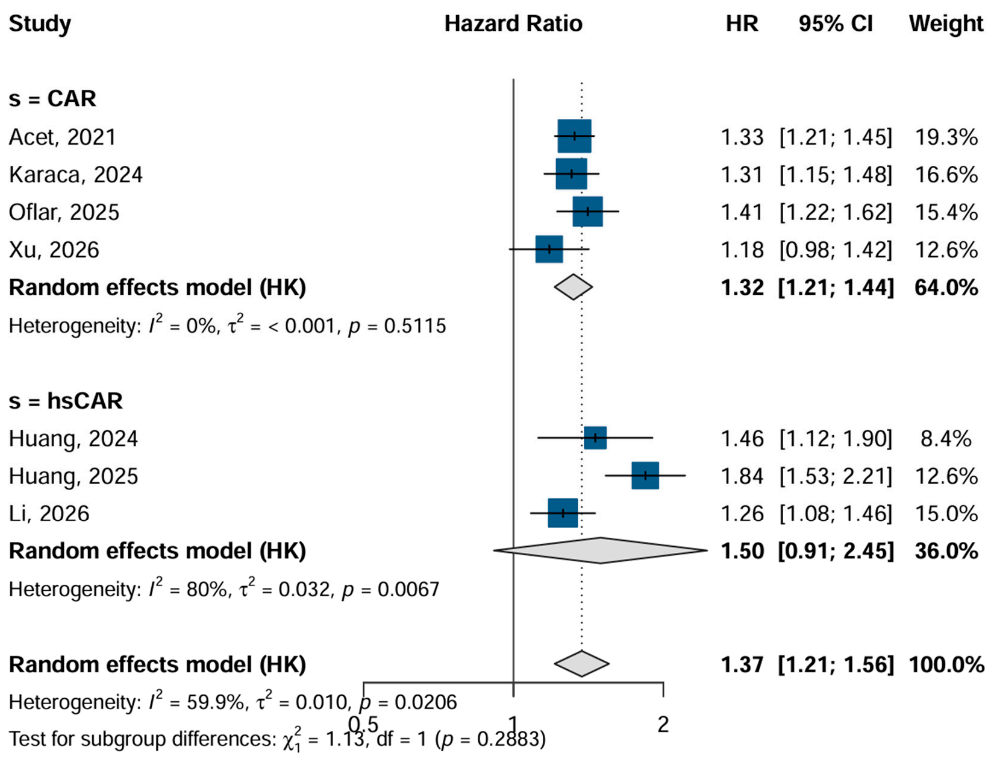

Figure S2. Subgroup analysis of NPAR and mortality by follow-up duration.

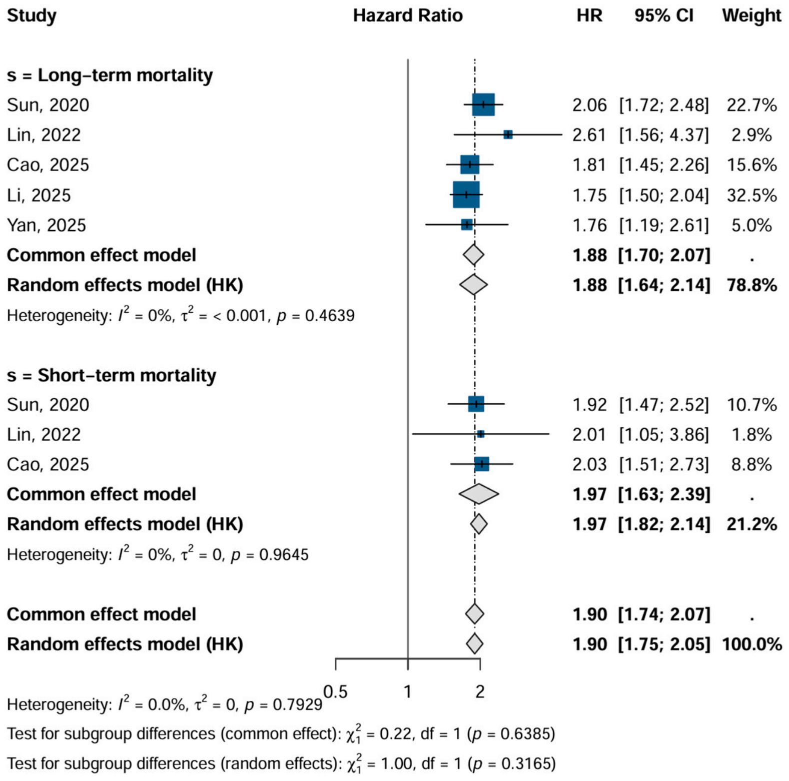

Figure S3. Subgroup analysis of the association between NPAR and mortality according to population type.

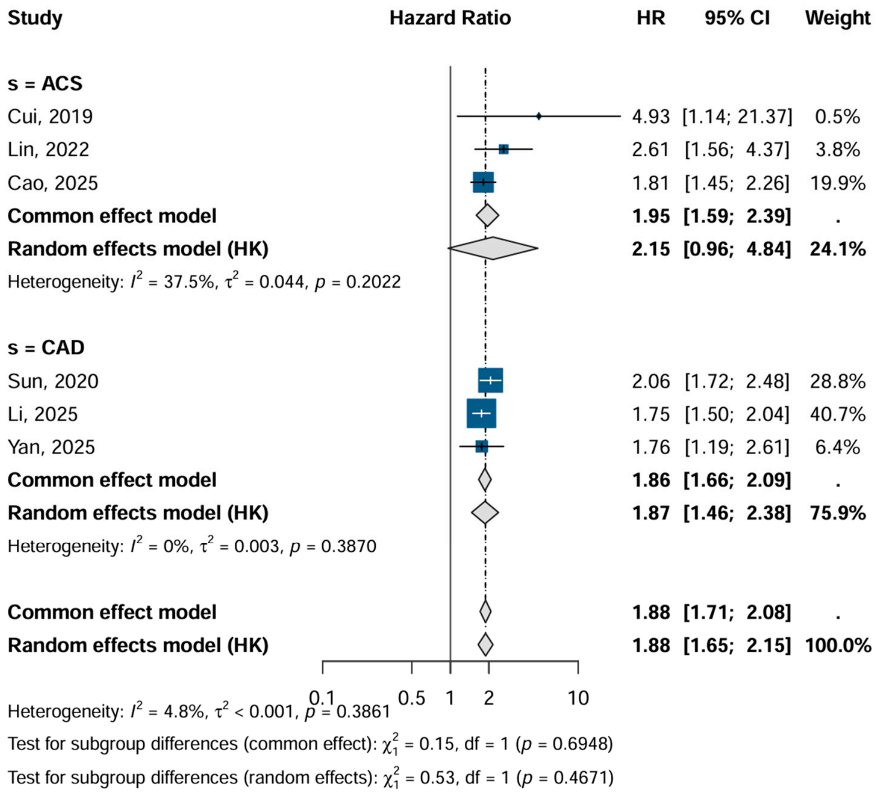

Figure S4. Association between LAR and mortality in ACS cohorts.

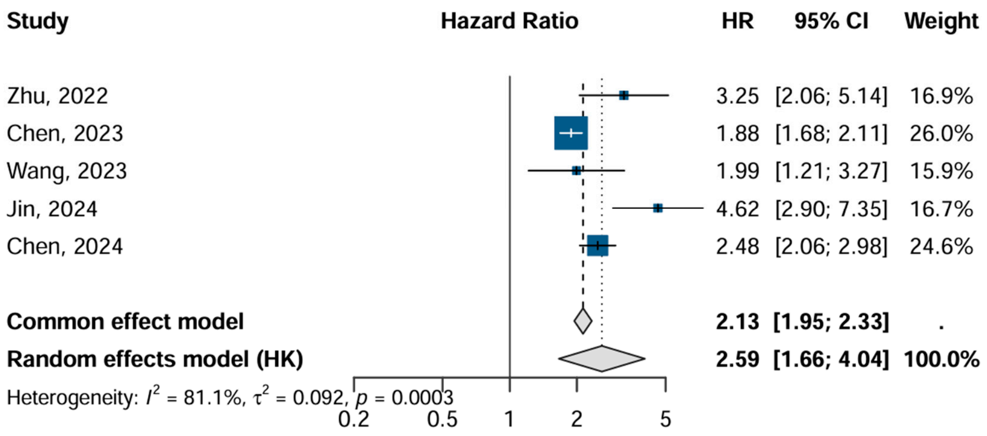

Figure S5. Discriminative performance of albumin-derived indices for predicting adverse outcomes in patients with cardiovascular disease. (A–C) Summary receiver operating characteristic (SROC) curves illustrating the predictive accuracy for long-term major adverse cardiovascular events (MACEs) of the C-reactive protein-to-albumin ratio (CAR) (A), neutrophil percentage-to-albumin ratio (NPAR) (B), and CALLY index (C).

(D–F) SROC curves showing the prognostic performance for all-cause and in-hospital mortality of CAR (D), CALLY index (E), and lactate-to-albumin ratio (LAR) (F).

(G–H) Comparative SROC plots showing differences in discriminative performance between mortality and long-term MACEs for CAR (G) and the CALLY index (H). In each panel, the solid curve represents the pooled SROC curve, the ellipse indicates the 95% confidence region, circles represent individual study estimates, and the diamond denotes the pooled summary point.

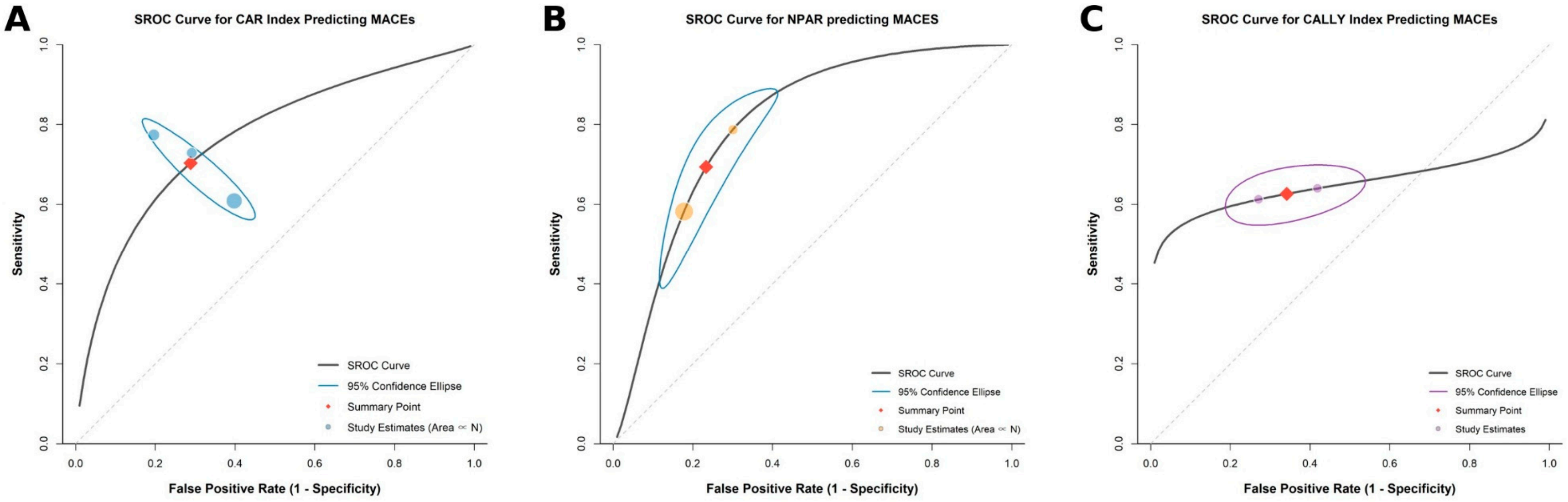

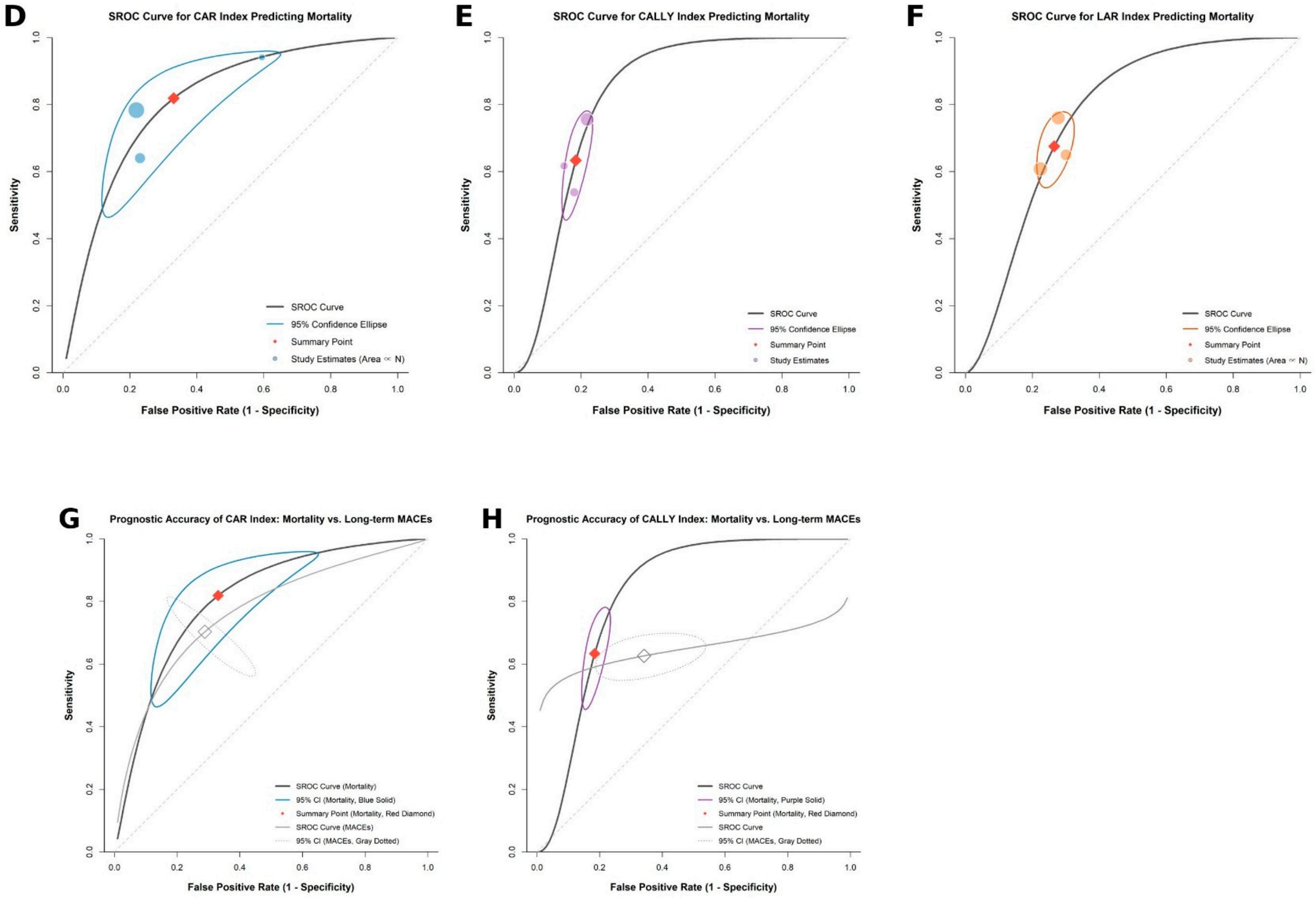

D
SROC Curve for CAR Index Predicting Mortality
Sensitivity
False Positive Rate (1 - Specificity)
SROC Curve
95% Confidence Ellipse
Summary Point
Study Estimates (Area ∝ N)
E
SROC Curve for CALLY Index Predicting Mortality
Sensitivity
False Positive Rate (1 - Specificity)
SROC Curve
95% Confidence Ellipse
Summary Point
Study Estimates
F
SROC Curve for LAR Index Predicting Mortality
Sensitivity
False Positive Rate (1 - Specificity)
SROC Curve
95% Confidence Ellipse
Summary Point
Study Estimates (Area ∝ N)
G
Prognostic Accuracy of CAR Index: Mortality vs. Long-term MACEs
Sensitivity
False Positive Rate (1 - Specificity)
SROC Curve (Mortality)
95% CI (Mortality, Blue Solid)
Summary Point (Mortality, Red Diamond)
SROC Curve (MACEs)
95% CI (MACEs, Gray Dotted)
H
Prognostic Accuracy of CALLY Index: Mortality vs. Long-term MACEs
Sensitivity
False Positive Rate (1 - Specificity)
SROC Curve
95% CI (Mortality, Purple Solid)
Summary Point (Mortality, Red Diamond)
SROC Curve
95% CI (MACEs, Gray Dotted)

Figure S6. Clinical utility of albumin-derived indices for predicting adverse outcomes.

(A–C) Fagan nomograms showing the clinical utility of CAR (A), NPAR (B), and CALLY index (C) for predicting long-term major adverse cardiovascular events (MACEs), using a pre-test probability of 20.0%. (D–F) Fagan nomograms showing the clinical utility of CAR (D), CALLY index (E), and lactate-to-albumin ratio (LAR) (F) for predicting all-cause and in-hospital mortality, using a pre-test probability of 10.0%.

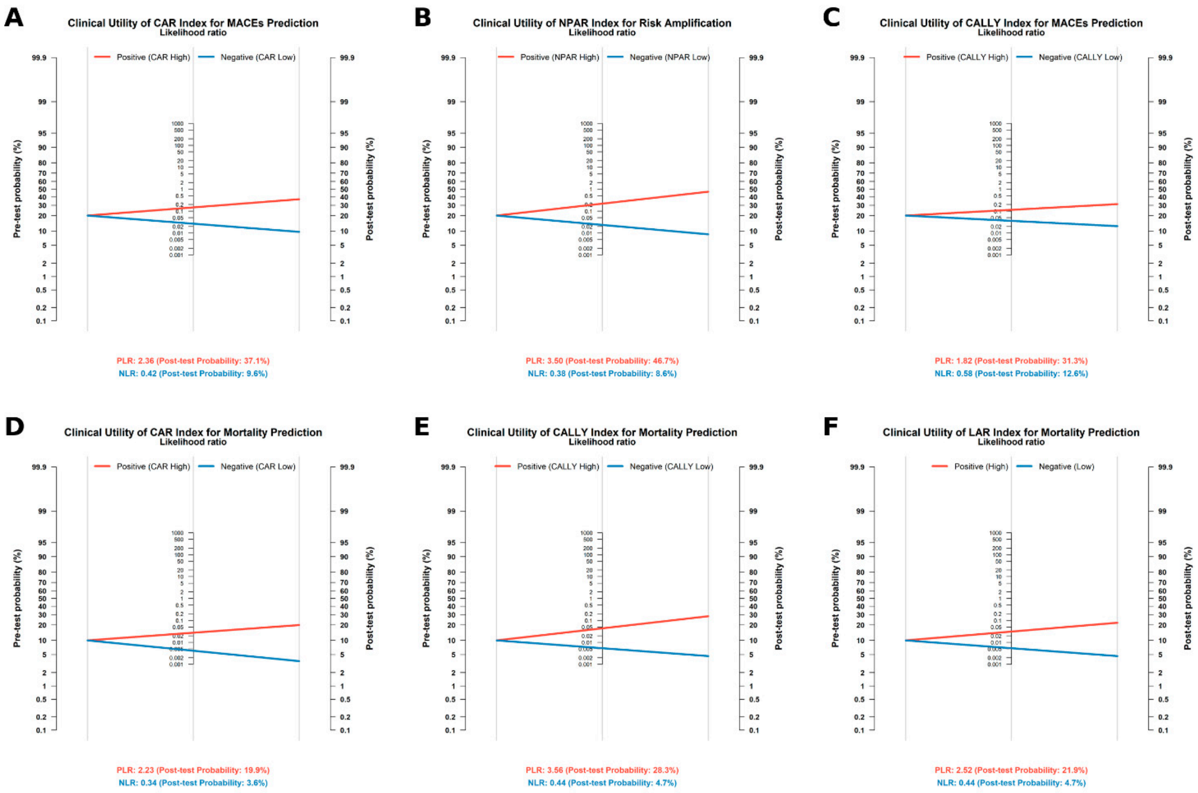

Figure S7. Sensitivity analyses of the main pooled outcomes.

Sensitivity Analysis (Leave-one-out)	I² (%)	Recalculated Effect (95% CI)
**A. CALLY Index and All-cause Mortality**		
Omitting Pan, 2024	46.7	0.58 (0.38 - 0.88)
Omitting Güven, 2025	73.1	0.49 (0.17 - 1.45)
Omitting Han, 2025	79.8	0.40 (0.13 - 1.25)
Omitting Wen, 2025	76.9	0.40 (0.14 - 1.12)
**Total (CALLY Mortality)**	**71.2**	**0.46 (0.24 - 0.87)**
**B. CAR/hsCAR Index and Mortality**		
Omitting Söğüt, 2021	80.7	2.00 (1.04 - 3.84)
Omitting Zhang, 2021	64.7	1.33 (1.06 - 1.65)
Omitting Birda, 2023	63.2	2.02 (1.11 - 3.67)
Omitting Menekşe, 2024	74.6	1.47 (1.07 - 2.02)
Omitting Arslan, 2025	73.0	1.90 (0.93 - 3.89)
**Total (CAR Mortality)**	**74.4**	**1.61 (1.09 - 2.38)**
**C. CAR/hsCAR Index and MACE**		
Omitting Acet, 2021	65.9	1.39 (1.18 - 1.63)
Omitting Huang, 2024	65.8	1.36 (1.17 - 1.59)
Omitting Karaca, 2024	65.8	1.39 (1.18 - 1.62)
Omitting Huang, 2025	0.0	1.32 (1.24 - 1.40)
Omitting Oflar, 2025	65.7	1.37 (1.17 - 1.60)
Omitting Li, 2026	63.9	1.39 (1.20 - 1.62)
Omitting Xu, 2026	60.3	1.40 (1.22 - 1.61)
**Total (CAR MACE)**	**59.9**	**1.37 (1.21 - 1.56)**
**D. NPAR Index and Mortality**		
Omitting Cui, 2019	0.0	1.88 (1.64 - 2.14)
Omitting Sun, 2020	0.0	1.82 (1.54 - 2.14)
Omitting Lin, 2022	0.0	1.86 (1.62 - 2.13)
Omitting Cao, 2025	21.5	1.92 (1.58 - 2.34)
Omitting Li, 2025	0.0	1.98 (1.66 - 2.37)
Omitting Yan, 2025	22.0	1.90 (1.60 - 2.26)
**Total (NPAR Mortality)**	**4.8**	**1.88 (1.65 - 2.15)**
**E. LAR Index and Mortality**		
Omitting Zhu, 2022	77.6	2.40 (1.59 - 3.64)
Omitting Chen, 2023	55.6	2.71 (1.83 - 4.02)
Omitting Wang, 2023	81.1	2.60 (1.70 - 3.98)
Omitting Jin, 2024	60.8	2.23 (1.75 - 2.84)
Omitting Chen, 2024	77.7	2.54 (1.59 - 4.05)
Omitting Liu, 2024	81.1	2.59 (1.66 - 4.04)
**Total (LAR Mortality)**	**76.5**	**2.50 (1.78 - 3.51)**

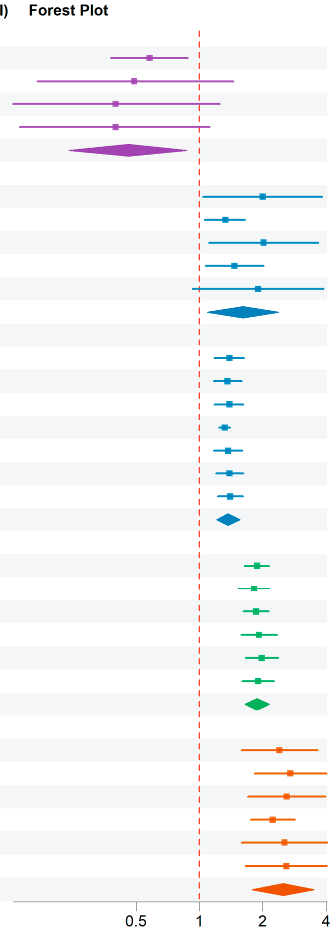

Supplement: Supplementary file 1 [file jcm-15-06868-s001.zip › jcm-4471018-supplementary.pdf]
